# Jaguar movement behavior: using trajectories and association rule mining algorithms to unveil behavioral states and social interactions

**DOI:** 10.1371/journal.pone.0246233

**Published:** 2021-02-04

**Authors:** Suelane Garcia Fontes, Ronaldo Gonçalves Morato, Silvio Luiz Stanzani, Pedro Luiz Pizzigatti Corrêa

**Affiliations:** 1 Computer Engineering and Digital Systems Department—Escola Politécnica da Universidade de São Paulo (USP), São Paulo, São Paulo, Brazil; 2 Centro Nacional de Pesquisa e Conservação de Mamíferos Carnívoros (CENAP), ICMBIO, Atibaia, São Paulo, Brazil; 3 Centro de Computação Científica, Universidade Estadual Paulista "Júlio de Mesquita Filho" (UNESP), São Paulo, São Paulo, Brazil; International Center for Theoretical Physics - South American Institute for Fundamental Research, BRAZIL

## Abstract

Animal movement data are widely collected with devices such as sensors and collars, increasing the ability of researchers to monitor animal movement and providing information about animal behavioral patterns. Animal behavior is used as a basis for understanding the relationship between animals and the environment and for guiding decision-making by researchers and public agencies about environmental preservation and conservation actions. Animal movement and behavior are widely studied with a focus on identifying behavioral patterns, such as, animal group formation, the distance between animals and their home range. However, we observed a lack of research proposing a unified solution that aggregates resources for analyses of individual animal behavior and of social interactions between animals. The primary scientific contribution of this work is to present a framework that uses trajectory analysis and association rule mining [Jaiswal and Agarwal, 2012] to provide statistical measures of correlation and dependence to determine the relationship level between animals, their social interactions, and their interactions with other environmental factors based on their individual behavior and movement data. We demonstrate the usefulness of the framework by applying it to movement data from jaguars in the Pantanal, Brazil. This allowed us to describe jaguar behavior, social interactions among jaguars and their behavior in different landscapes, thus providing a highly detailed investigation of jaguar movement decisions at the fine scale.

## Introduction

In recent years, several studies have revealed important aspects of jaguar (*Panthera onca*) movement ecology. In general, these studies are focused on understanding large-scale ecological processes, such as space use and habitat selection, and highlight jaguar responses to habitat and human activity variables [1–5]. Moreover, there are still some knowledge gaps regarding movement behavior at a fine scale, such as movement related to behavior states and social interactions.

Human activities have impacted the movement behavior of several mammal species; for example, carnivore species, such as the jaguar, have had to move longer distances to find mates and obtain resources [4–6]. While moving, animals may change their movement behavior state or spend time socially interacting. Identifying behavioral changes and social interactions are crucial aspects of species ecology. In the first case, the transition from resting to foraging or traveling may contribute to investigating animals’ responses to either biotic or abiotic factors [7] at a fine scale. For example, what is the jaguar’s foraging strategy in harsh environmental conditions or in an agricultural landscape? In high human density areas, does the jaguar rest during the day and forage and travel during the night? In the second case, interactions may result in competition, predation, reproduction or disease transmission [8], which can also be related to habitat conditions. For example, in areas of high primary productivity, jaguars are found at high densities [9], and we may expect greater spatiotemporal overlap with more instances of mating and/or competition.

The jaguar social system is based on territoriality, with adults maintaining individual territories [10, 11]. The use of space by animals is directly influenced by environmental factors, such as the availability of resources and the presence of other animals. Space sharing by animals provides diverse and valuable information about disease transmission, social behavior and gene flow [12]. Spatial overlap has been described among males, females, and male-female pairs [1, 13]; however, until recently, the description of spatiotemporal interactions was restricted to individual events registered by camera traps [14] or visualization. Using step selection functions [15] were the first to incorporate the spatiotemporal dynamics of conspecific movements; however, that study focused only on animal interactions. The interaction of large mammals also was studied by others [16, 17], but no study did address the use of data mining techniques to analyze the social interaction between typically solitary animals such as the jaguars.

Data mining, in data science, is the stage of data analysis that comprises the set of strategies, tools and algorithms that allow data exploration and the extraction of data patterns [18]. The ObjectGrowth method [19], for example, is a proposed method of discovering closed swarms using density-based clustering data mining with the DBSCAN algorithm [20] to generate animal groups and the Monte Carlo method to calculate the significance of relationships. ObjectGrowth [21] and Periodica [21] together form MoveMine [22], a motion data mining system for behavior discovery. ObjectGrowth extracts swarm patterns using density grouping with DBSCAN, and Periodica detects periodic behavior. The Get move algorithm [23] also uses DBSCAN [20], to discover movement patterns, closed swarms, convoys and group patterns. Clustering data mining techniques divide the data into groups of similar objects; it has also been applied to identify patterns of flock behavior, i.e., a set of animals that move together for a given continuous period [24] and is used in the discovery of interactions between animals but focuses on identifying groups and does not provide statistical measures about the interactions. On the other hand, association rule mining provides statistical measures of correlation and dependency between the associations.

The Apriori [25], an association rule mining algorithm, was used to find movement patterns of birds and species with periodic collective movement [26]. Association rule mining is a method for discovering interesting relationships between variables in large data sets [27]. Association rules have been used in several applications to find frequent patterns in the data, mainly in the discovery of relationships between products in market basket analysis [28] to guide marketing actions and provides statistical measures of correlation and dependency between the associations.

Animal movement and behavior are widely researched, encompassing studies about the discovery of animal behavioral patterns to determine the distance between animals, trajectory segmentation, classification of animal behavior and mapping of the animal home range. However, we have observed that such research generally addresses a specific aspect of animal behavior or identifies animal groups but does not provide statistical measures about the interaction level between them and is limited to analysis about the interactions between animals and other environmental factors.

In this study, we proposed a framework that uses trajectory analysis and association rule mining [27] to provide statistical measures of correlation and dependence between associations and can be used to determine the relationship level between animals, their social interactions, and their interactions with other environmental factors based on their individual behavior and movement data. The higher the frequency of cooccurrence of these animals is, the greater the likelihood of interaction between them. In addition, the framework allows us to analyze individual animal behavior by classifying the behavior into states (rest, transit and foraging) and to obtain valuable information about the duration, frequency, periods (day/night) and locations of the animal behavioral states.

We conducted a case study applying the framework developed to movement data from jaguars tracked in the Pantanal, Brazil. The aim was to provide insights into jaguar behavioral transitions over time and space and about the social interactions between jaguar pairs and their behavior in different landscapes, thus contributing to a better understanding of the species’ movement decisions at a fine scale.

## Materials and methods

### Data

We investigated the movement behavior of nine jaguars inhabiting the Taiamã Ecological Station, Pantanal, Brazil. The animals were fitted with GPS-satellite collars (Lotek Wireless, Inc.) and monitored for periods of 60 to 591 days (permit: SISBIO#30896–1) [5]. The authors of the study own the data set [5] and made it public and freely available at DOI: 10.1002/ecy.2379 and also at Dryad Digital Repository (https://doi.org/10.5061/dryad.2dh0223).

The land cover data set from Taiamã was extracted from the MapBiomas Project—Collection 4.1 of the Annual Series of Coverage and Land Use Maps in Brazil [29], accessed freely on 06/07/20 through the link: https://mapbiomas.org. We authors did not have any special access to the data. This project is a multi-institutional initiative to generate annual maps of land cover and use from automatic classification processes applied to satellite images.

Algorithms and minimal datasets, results (tables and figures) and reproducible code are available in a GitHub repository: https://github.com/suelanegarcia/AniMoveMiner/tree/master/Jaguars_Taiama_PlosOne

### Trajectories and association rule mining algorithm framework

We present a framework that uses trajectory analysis and association rule mining [27] to provide statistical measures of correlation and dependence to determine the relationship level between animals, their social interactions, and their interactions with other environmental factors based on their individual behavior and movement data.

**Fig 1:**
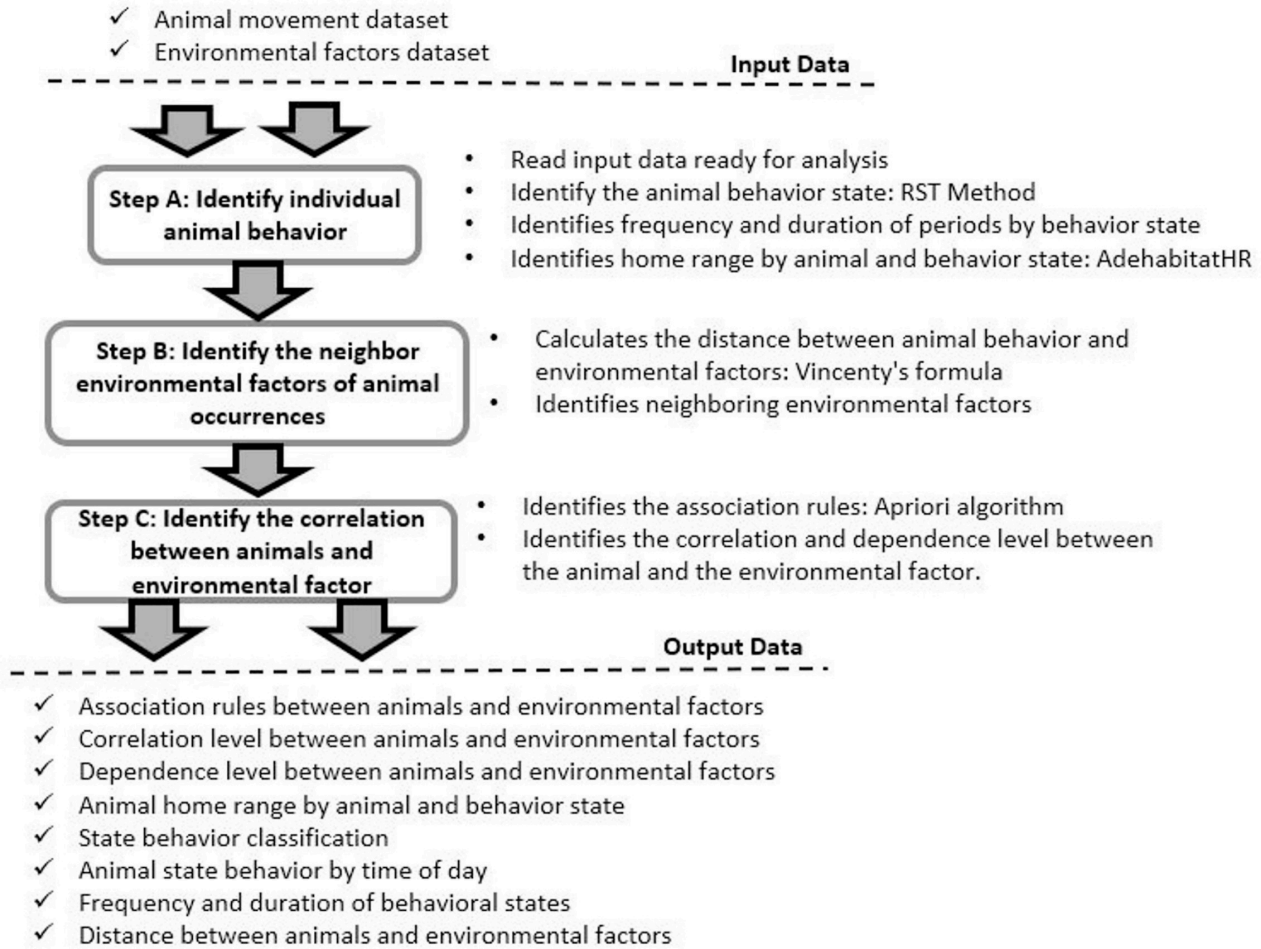
Framework steps. The framework is composed of three steps: (A) identify the behaviors of individual animals; (B) identify the influence of neighboring animals on the animal occurrences; and (C) identify the correlation between animal behavior and environmental factors. The framework receives animal movement data and environmental factors as input. First, the animal movement data are analyzed to identify the individual behavior of the animals. Then the environmental factors that occurred close to the animals are identified. In the last step, the interactions between animals and between animals and other environmental factors are identified. Each step provides information about the animals and their interactions that are presented in tables, graphs, or maps.

To use the strategy, we provided as an input spatiotemporal data describing the movement of one or more animals, characterized by tuples of historical movement data composed of location points (latitude/longitude), times (date/time) and animal names. The steps of our strategy followed data science processes [30], considering that the process began by formulating the questions to be answered and included data acquisition from data sources and data preparation for analysis. The data analysis step is the main focus of this paper, in which the analysis of animal behavior is divided into three phases (Fig 1): (A) identify the behaviors of individual animals; (B) identify the influence of neighboring animals on the animal occurrences; and (C) identify the correlation between animal behavior and the surrounding environmental factors.

### Identifying the correlations between animal behavior and the presence of neighboring animals

In the analysis of the interactions between jaguars, we applied the association rule mining technique using the Apriori algorithm in the Arules package [31] to obtain information about the cooccurrence of jaguar pairs within a given radius and time window. The Apriori algorithm [31] finds elements that imply the presence of other elements in the same data set and indicates the cooccurrence frequency of the elements. The algorithm strategy consists, first, of determining the set of frequent items that have support greater than or equal to the minimum support (minSup). The minimum support indicates the minimum frequency one or more of the items occur together. Second, the algorithm generates the association rules, keeping only those with confidence greater than or equal to the minimum confidence (minConf). The minimum confidence indicates minimum probability that an item will occur, given the occurrence of another item. The minSup and minConf parameters are used by the Apriori algorithm to limit the number of association rules extracted and to validate the data set, making it possible to discard less-relevant and less-frequent rules. The lower the minSup value, the lower the frequency of cooccurrence between the variables contained in the rules that are considered relevant in the analysis. The lower the minConf value, the lower the probability that one variable will occur due to the other. In the jaguar interactions analysis, we identified the association rules between variables, for example, Jaguar A and Jaguar B, based on the cooccurence frequency of them and removed the rules that were below the limits of minSup and minConf.

The Apriori algorithm generates as a result the association rules between the variables and several interestingness measures. These measures encompass information about the occurrence frequency, correlation, and dependency between the analyzed variables. Our approach considered four of these measures in the analysis of the jaguars: support (Sup), confidence (Conf), lift and correlation coefficient (Phi).

Support (Sup) shows the probability of the jaguars cooccurrence in a specific radius and time window and indicates the relevance of the rule,

$$Support\;(Sup)=\frac{Total\;occurrence\;(A\cap B)}{Total\;occurrences\;(D)}$$ (1)

where the total number of cooccurrences of jaguar A and jaguar B are divided by the total occurrences contained in the data set (D).

Confidence (Conf) shows the probability of a variable, for example, Jaguar B, occurring due to the occurrence of another, Jaguar A, and indicates the validity of the rule,

$$Confidence\;(Conf)=\frac{Total\;occurrence\;(A\cap B)}{SupA}$$ (2)

where the total number of cooccurrences of jaguar A and jaguar B are divided by the total number of occurrences of jaguar A, calculated as:

$$SupA=\frac{Total\;occurrence\;of\;A}{Total\;occurrences\;(D)}$$ (3)

where the total number of occurrences of jaguar A are divided by the total occurrences contained in the dataset (D).

Lift indicates the dependence between jaguars, indicating the influence that the occurrence frequency of one jaguar has on the occurrence of the other. The dependence was classified as follows: positive, with a value of 1, which indicates that the presence of one jaguar increases the occurrence of the presence of another jaguar; negative, with a value of -1, which indicates that the presence of one jaguar decreases the occurrence of the presence of another jaguar; and independent, with a value of 0, which indicates that one jaguar does not influence the presence of another jaguar. The lift is calculated as:

$$Lift=\frac{Conf}{SupB}$$ (4)

where Conf represents the probability of Jaguar B occurring due to the occurrence of Jaguar A divided by SupB,

$$SupB=\frac{Total\;occurrences\;of\;B}{Total\;occurrences\;(D)}$$ (5)

where the total occurrences of jaguar B are divided by the total occurrences contained in the data set (D).

The correlation coefficient (Phi) indicates the degree of the relationship, i.e., the correlation level, between the jaguar pair (Jaguar A and Jaguar B) based on cooccurrence between them. Phi is calculated as:

$$Phi=\surd \frac{Sup-(SupA)(SupB)}{\left(SupA)(SupB)(1-SupA)(1-SupB\right)}$$ (6)

The Phi classifications include the following: perfect negative correlation (value of -1); strong negative correlation (values from -1 to -0.68); moderate negative correlation (values from -0.67 to -0.36); weak or no correlation (values from -0.35 to 0.35); moderate positive correlation (values from 0.36 to 0.67); strong positive correlation (values from 0.67 to 1); no correlation (value of 0); and perfect positive correlation (value of 1). Our approach used the Cramer coefficient to validate the correlation analysis results obtained by association rule mining. This coefficient indicates the association between two categorical variables and was calculated based on the chi-square value [32] obtained in the contingency table analysis. The result ranges between 0, representing total independence, and 1, representing total association; the stronger the association is, the higher the coefficient value.

### Identifying individual animal behavior

An analysis of jaguar movement data was conducted to obtain knowledge about the individual behavior of each animal, including the identification of each jaguar’s behavioral states, the frequency and duration of these states and their home range. Individual behavior analysis identifies what the animal is doing, where and when [33].

In the animal behavioral state classification, adehabitatLT [34] was used to define and manipulate the trajectories, and the RST method [35] was employed to classify animal behavior according to the state (rest, transit and foraging) by segmenting the trajectories based on the amount of space and time occupied by the animal in an area. Around each point of the trajectory, a circle was constructed, and the following were calculated: the distance traveled (RD), which refers to the sum of the lengths of the path within the circle, and the residence time (RT), which refers to the sum of the residence times between consecutive points within the circle. RD and RT values were normalized by dividing by the maximum respective value within each track. The difference between RD e RT is calculated and obtain the residual result (between -1 and 1). The states are classified as follows: transit (residual = 0), a behavior with a short duration and distance covered in the area; rest (residual < 0), a behavior with a long duration but a short distance covered in the area; and foraging (residue > 0), a behavior with a long duration and a large distance covered in the area.

#### Identifying the frequency, duration, and period of the occurrence of the animal behavior states

A routine in R was used in the framework to analyze the jaguar movement data to identify periods (day/night), times and average duration of the occurrence of animal behavioral states. The results of the following distributions were obtained: animal behavioral states in the time intervals (start time and end) over the days, months and years; the average duration time of each behavioral state by month and year; and the state occurrence frequency by period (day/night) for each animal over the months and years.

#### Identifying the home range

The home range is defined as the probability that an animal is found at a given point according to its geographical coordinates, which is called a utilization distribution (UD). The function kernelUD [36] in the adehabitatHR package was applied to estimate the utilization distribution of the space by animals. Basically, the kernel method applies a bivariate normal distribution around each location and sums these distributions over the landscape.

The core area of the home range, which is used more often by an animal, is considered the minimum area in which an animal has a specified high probability of being located. The function kerneloverlap [36] was used to identify the area overlap between the animals. Exploring the home range provides information about how the jaguars occupied and shared the space over time.

#### Identifying the surrounding environmental factors related to animal occurrences

Our approach considers that the closer an animal behavior occurs to an environmental factor, the greater the likelihood that there is a relationship between the behavior and the factor. Therefore, to identify factors related to neighboring animals when these factors contain information about space (latitude/longitude) and time, an array was created to sort the data according to the date and time of occurrence, and the distance between location points was calculated using the Vincenty formula [37]. This formula calculates the distance, or shorter curve, between pairs of points on the Earth’s surface considering the flattening of the poles. For each record of animal movement, a circle with a limited-distance radius was created around the point (position and time), and the distance between the points of the movement and the factor was calculated. The presence of other jaguars was considered an environmental factor, so the distance between the jaguars was calculated using the Vincenty formula.

## Results

The results obtained at each step of the framework in the jaguar data analysis allow us to explore aspects of individual animal behavior, the interactions between animals over time and space and the interactions between the animal behavior state and the land cover types. The analysis of jaguar movement provides insight into individual jaguar behavior that indicates the behavioral states (foraging, rest and transit) along their trajectories, the frequency, duration, periods (day/night) and times of the occurrence of these behavioral states, and the area occupied by the jaguars (home range). The results are provided as a map referring to the region tracking of jaguars and containing the individual trajectories of jaguars over time, allowing a comparison with the trajectories of other animals throughout the months of the year.

**Fig 2:**
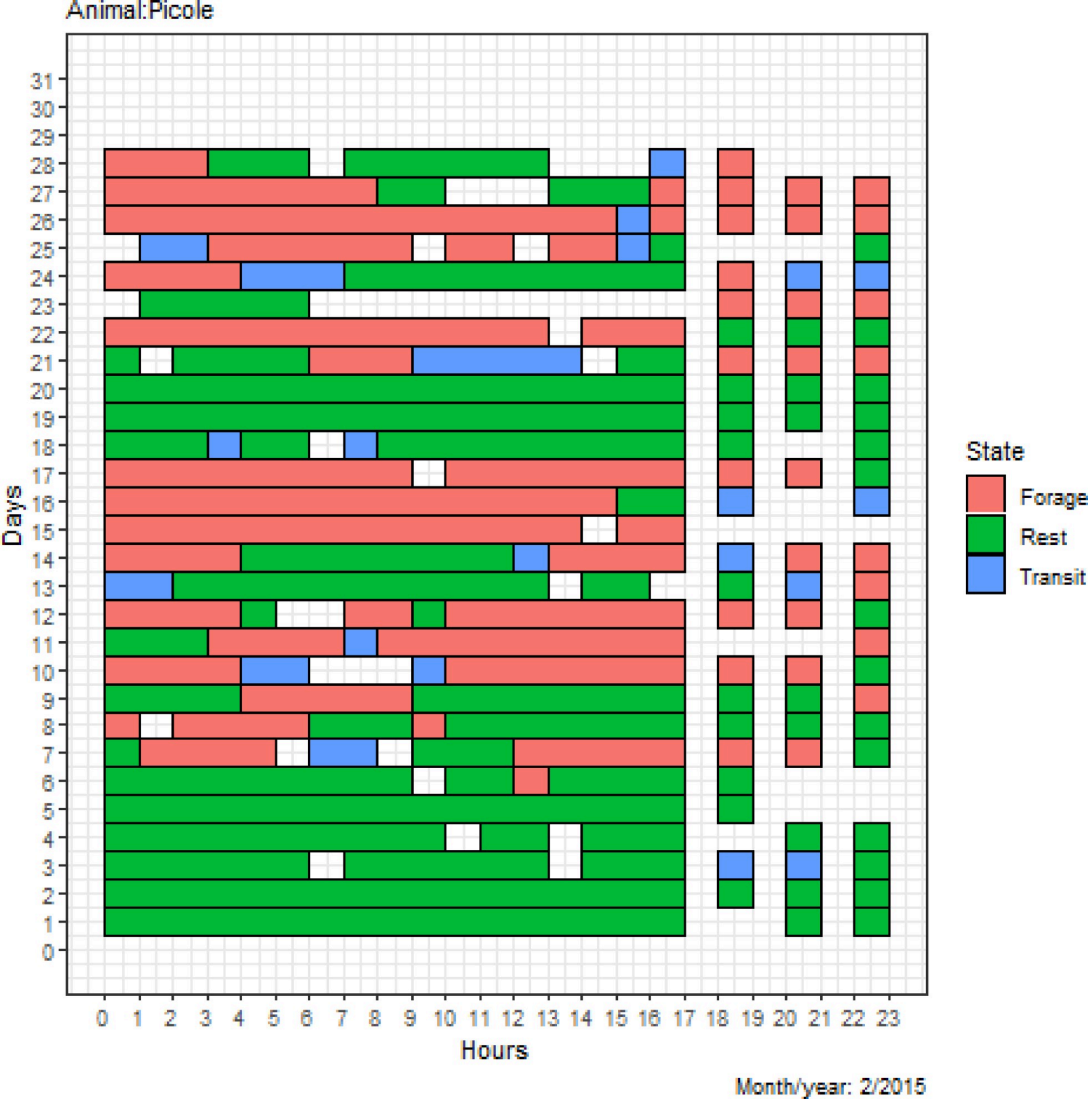
Behavioral state matrix of the jaguar Picolé. The matrix shows the time intervals that the jaguar Picolé spent foraging, resting or in transit over the study period. Picolé presented a higher frequency of the resting state in the first days of February, and on days 10, 11, 16 and 17, there was a higher frequency of foraging. This distribution identifies the individual behaviors of the animal in its daily activities, as well as the changes in animal behavior over the days and months of the year.

A matrix of each jaguar’s behavioral state was obtained as shown in Fig 2, which shows the distribution of states in hourly intervals over the days of each month/year. The matrix in Fig 2 makes it possible to observe the time intervals that the jaguar Picolé spent foraging, resting or in transit over the study period. For example, Picolé presented a higher frequency of the resting state in the first days of February, and on days 10, 11, 16 and 17, there was a higher frequency of foraging. This distribution allows us to identify the individual behaviors of the animal in its daily activities, as well as the changes in behavior over the days and months of the year.

Another aspect of individual jaguar behavior that is provided by the framework is the duration of the behavioral states, which allows us to identify the average amount of time the jaguar spends on its foraging, resting and transit activities. A graph shows the average duration of each state of behavior for the jaguar Picolé throughout the months of the year, indicating the trend of increasing or decreasing duration as a function of the month of the year. Picolé, as shown in S1 Fig, spent an average duration of two hours in the foraging state in the months of January to April, which decreased to one hour in March 2015.

The analysis also identifies the occurrence periods of animal behavior states and indicates the times at which these states occur most frequently. The graph in S2 Fig shows the intervals between hours at which the foraging state occurred the most frequently over the months of 2015 for the jaguar Picolé; the circle represents the start time and the line indicates the duration, thus demarcating the beginning and end of the period. The results show the months from January to May 2015 and indicate that Picolé foraged more frequently during the hours from 0 a.m. to 8 a.m., with some variations over the months.

By detailing the times of occurrence of animal behavioral states, the frequency at which states occurred throughout the periods of the day (day/night) was identified. The graph in S3 Fig shows the occurrence frequency of each of Picolé’s states of behavior over the months from January to May 2015, indicating a higher number of foraging occurrences at night in February and March, as well as an increase in occurrences followed by a decline in the months of April and May 2015.

The jaguar’s home range was also analyzed, which allowed us to verify how the space was occupied by the jaguar over the months of the year based on its behavioral states. The map in S4 Fig provides information about the variation in the space occupied by the animal over the months and years. The jaguar Picolé, for example, occupied a certain area in January 2015, migrated over February to a new area and remained in this area throughout the following months.

This analysis provides valuable information for answering questions about the individual jaguar’s behavior, such as: How much time does the jaguar spend foraging? What times does jaguar usually forage or rest? Does the jaguar forage more during the day or night?

In the analysis of the interactions between jaguars, to identify the neighboring animal, the distance limit radius were defined as <200 m, which suggests a certain degree of sociability [1], and as <400 m, to verify the results of the framework with the variations in the distance limit. In both cases, the distance between jaguars was estimated using the Vincenty formula.

Based on these data, the Apriori algorithm was applied to perform association rule mining to identify the relationships between the animals. The evaluations used varying values for the parameters minSup and minConf, defined as 0.01 (1%), 0.50 (50%) and 0.9 (90%), to filter out the most relevant rules for the analysis.

For the different tests performed, the mining by association rules for the jaguar movement data provided the measurements shown in Table 1: support, confidence, lift and the phi coefficient. In the results obtained with the distance limit as <200 m, the support measure provided the variation in the cooccurrence between the jaguar pairs over the months. For February, 75% of the cooccurrences were identified as being for the jaguar pair of Caiman and Dale and 25% as being for Caiman and Fera. Therefore, Caiman and Dale presented a high frequency of cooccurrence. The confidence measure, which determines the probability of one animal occurring as a function of the occurrence of the other, also indicated a strong relationship between Caiman and Dale in February.

**Table 1:** Measures resulting from mining by association rules between jaguars for <200 distance.

Jaguars	Month	SupA	SupB	Sup (%)	Conf (%)	Phi (%)	Lift	Occurrences (A∩B)
Caiman/Dale	February	1	0.75	75	75	NA	1	3
Caiman/Fera	February	1	0.25	25	25	NA	1	1
Dale/Caiman	February	0.8	1	75	100	NA	1	3
Dale/Fera	March	1	1	100	100	NA	1	2
Dale/Fera	April	1	1	100	100	NA	1	5
Dale/Fera	May	1	1	100	100	NA	1	33

In March, April and May 2015, the jaguars Fera and Dale did not cooccur with Caiman but had a 100% occurrence probability due to the occurrence of the other (support and confidence). In these months, a strong cooccurrence between the jaguars Dale and Fera was identified, indicating a higher relationship between these jaguars for all evaluated minConf and minSup values, including the value equal to 90%.

However, we observed that for all results, the phi coefficient was equal to NA, and the lift is equal to 1 (Table 1). This occurs when the support and confidence values are equal because the total occurrences of the jaguars together are the same as the probability of one occurring when the other occurs in the data set. In these cases, it is necessary to consider more relevant support and confidence measures and validate the results using the Cramer coefficient and overlapping home range.

At the distance limit <400 m, as shown in (Table 2), we observed a relationship between the jaguars Alice and Picolé in February due to the increase in the distance limit. The phi and lift of this analysis were more relevant than those in the previous analysis and indicated correlation and dependence levels.

**Table 2:** Measures resulting from mining by association rules between jaguars for <400 distance.

Jaguars	Month	SupA	SupB	Sup (%)	Conf (%)	Phi (%)	Lift	Occurrences (A∩B)
Alice/Picolé	February	0.375	0.375	38	100	1	2.666	3
Caiman/Dale	February	0.625	0.375	38	60	0.6	1.6	3
Caiman/Fera	February	0.625	0.25	25	40	0.447	1.6	2
Dale/Fera	March	1	1	100	100	NA	1	5
Dale/Fera	April	1	1	100	100	NA	1	21
Dale/Fera	May	1	1	100	100	NA	1	63

The correlation between animals was also analyzed through the contingency table and Cramer coefficient to complement and validate the results obtained with the association rules. The results were presented by month and year and indicated a Cramer coefficient equal to 0.707, relatively close to 1, for February 2015, which indicates correlations between the jaguars Caiman and Dale and between Caiman and Fera (Table 3). The results showed a higher correlation between Caiman and Dale, which is in line with the association rules in which Caiman and Dale presented 75% cooccurrence and Caiman and Fera presented 25% cooccurrence (Table 1). For March, April and May 2015, the Cramer coefficient was equal to 1, indicating a strong correlation between the jaguars Dale and Fera. The correlation between the jaguars obtained with the association rules was confirmed using the contingency table and the Cramer coefficient.

**Table 3:** Cramer coefficient results at <200 m distance.

Jaguars	Month	Occurrences	Cramer’s V
Caiman/Dale	February	2	0.707
Caiman/Fera	February	1	
Dale/Fera	March	3	1
Dale/Fera	April	3	1
Dale/Fera	May	3	1

**Fig 3:**
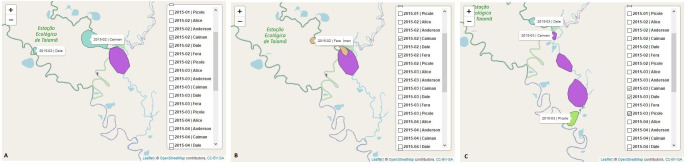
Home range maps to the jaguars Caiman, Dale and Fera. The graph shows the use of space by these jaguars over the months of February and March 2015. The kernel function [38] was used to calculate the home range of the jaguars in space and time and to determine the minimum area in which each animal had a high probability of being located. The results indicated that Caiman and Dale [A] and Caiman and Fera [B] presented home range overlap in February 2015. In March 2015, Caiman distanced itself from Dale and approached Picolé [C]. The home range overlap provides information about the social interactions between jaguars.

The interactions between jaguars can also be observed through the home range overlap. In the framework, the home range overlap can be explored using the map showing the area occupied by the jaguars by month and year or by month, year, and behavioral state. The space used by the jaguars Dale, Fera and Caiman was identified, and it was observed that Caiman and Dale Fig 3[A] and Caiman and Fera Fig 3[B] presented home range overlap in February 2015, but in March 2015, Caiman distanced itself from Dale and approached Picolé Fig 3[C].

**Fig 4:**
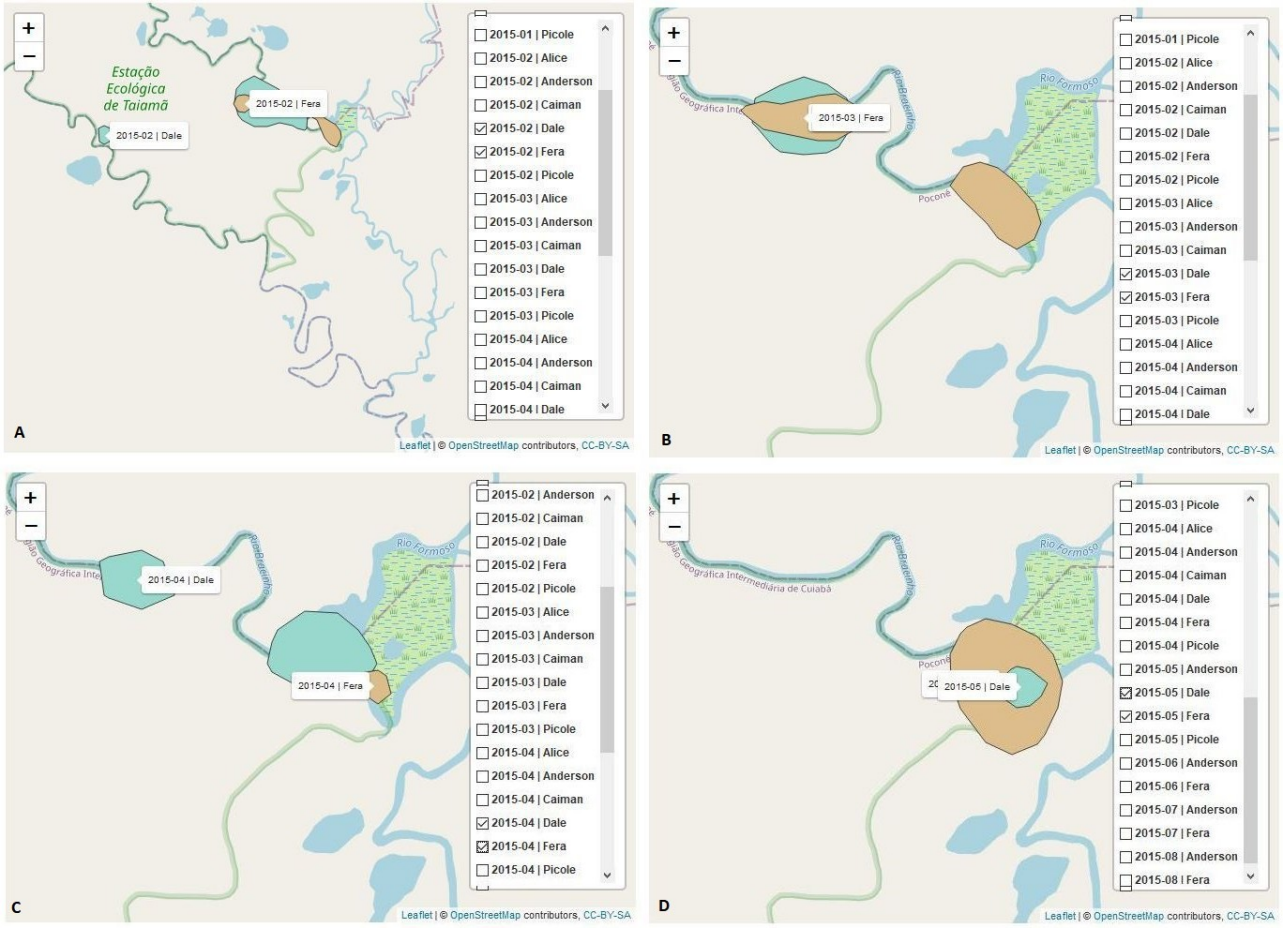
Home range maps of the jaguars Dale and Fera. The graphs show the use of space by these jaguars over the months of February, March, April, and May 2015. The kernel function [38] was also used to calculate the home range of the jaguars in space and time and to determine the minimum area in which each animal had a high probability of being located. Dale and Fera presented home range overlap in all analyzed months in 2015: February [A], March [B], April [C] and May [D]. The home range overlap indicates a high frequency of interaction between these two jaguars.

Dale and Fera presented home range overlap in all analyzed months: February Fig 4[A], March Fig 4[B], April Fig 4[C] and May 2015 Fig 4[D].

The framework provides the home range of the jaguars based on their behavioral state and movement data over the months of the year. In this way, home range overlap analysis can be used to confirm the results obtained with the association rules to identify encounters between jaguars and to track the behavioral changes in these jaguars with respect to their use of space over time.

Another aspect of the interactions between animals that can be analyzed is the distance between them over time. A distance limit radius for identifying neighboring animals was defined, and the analyses provided the day, month, and year that the jaguar pairs were registered at a distance less than the distance limit radius.

The results indicated that Dale and Fera had the highest number of cooccurrences within the distance limit of <200 m within the study period, while the pairs Caiman and Dale and Caiman and Fera occurred just one time each (Table 4).

**Table 4:** Distance between jaguar pairs at the distance limit < 200.

Jaguars	Date/Time	Distance (km)
Caiman/Dale	2015-02-22 15:01:42	0.011
Caiman/Fera	2015-02-25 22:00:33	0.019
Fera/Dale	2015-03-20 12:00:41	0.111
Fera/Dale	2015-03-26 13:00:34	0.193
Fera/Dale	2015-04-06 22:00:23	0.176
Fera/Dale	2015-04-19 00:00:20	0.030
Fera/Dale	2015-05-05 14:00:17	0.043
Fera/Dale	2015-05-06 00:00:29	0.016
Fera/Dale	2015-05-07 01:00:48	0.022
Fera/Dale	2015-05-08 10:00:19	0.197
Fera/Dale	2015-05-10 15:00:13	0.026
Fera/Dale	2015-05-15 20:00:12	0.016

In addition to the analysis of social interaction between jaguars, we analyzed the interaction between the states of jaguar behavior (rest, forage, and transit) and the type of land cover. Association rule mining was applied, and the results indicated the correlation and dependence level between the jaguar behavior state and the land cover at the Taiamã Ecological Station (Table 5).

**Table 5:** Measures resulting from mining by association rules between forage state and land cover.

Animal	Behavior state/Land Cover	SupA	SupB	Sup (%)	Conf (%)	Phi (%)	Lift	Occurrences (A∩B)
Alice	Forage/Wetland	0.133	0.011	0	3	5	2	7
Picolé	Forage/Wetland	0.352	0.061	5	14	25	2	124
Picolé	Forage/Water	0.352	0.015	0	1	-1	1	11
Picolé	Forage/Forest	0.352	0.070	1	2	-13	0	21
Fera	Forage/Wetland	0.198	0.098	3	16	11	2	146
Fera	Forage/Water	0.198	0.011	1	3	7	2	24
Fera	Forage/Forest	0.198	0.890	16	81	-12	1	736
Caiman	Forage/Wetland	0.185	0.188	5	28	12	2	88
Caiman	Forage/Water	0.185	0.018	1	3	5	2	10
Caiman	Forage/Forest	0.185	0.745	11	58	-18	1	181
Dale	Forage/Water	0.101	0.012	0	3	5	2	9
Dale	Forage/Wetland	0.101	0.050	1	5	0	1	16
Dale	Forage/Forest	0.101	0.936	9	91	-3	1	276
Anderson	Forage/Wetland	0.231	0.094	3	12	5	1	126
Anderson	Forage/Water	0.231	0.023	1	3	1	1	27
Anderson	Forage/Forest	0.231	0.881	20	85	-5	1	900

The results showed a higher probability of correlation and dependence between the foraging state and the wetland with the jaguars Alice, Picolé, Fera, Caiman and Anderson. Only the jaguar Dale showed a greater correlation and dependence between the foraging state and water. Based on the results, the jaguars forage more often in the wetland than in other land cover types in the study area.

The results showed a high probability of resting in the forest for all jaguars (Table 6). Negative correlations between the resting state and wetlands and water for the jaguars Alice, Fera, Caiman, Dale and Anderson were also observed. Based on the results, jaguars are more likely to rest in areas of forest than in other land cover types in the region.

**Table 6:** Measures resulting from mining by association rules between rest state and land cover.

Animal	Behavior State/Land Cover	SupA	SupB	Sup (%)	Conf (%)	Phi (%)	Lift	Occurrences (A∩B)
Alice	Rest/Forest	0.790	0.015	1	2	1	1	26
Alice	Rest/Wetland	0.790	0.011	1	1	-4	1	15
Picolé	Rest/Forest	0.575	0.070	6	10	15	1	145
Picolé	Rest/Water	0.575	0.015	1	2	3	1	25
Picolé	Rest/Savanna	0.575	0.002	0	0	1	1	4
Picolé	Rest/Wetland	0.575	0.061	1	1	-25	0	13
Fera	Rest/Forest	0.773	0.890	71	92	18	1	3259
Fera	Rest/Water	0.773	0.011	0	1	-10	0	19
Fera	Rest/Wetland	0.773	0.098	6	7	-15	1	262
Caiman	Rest/Forest	0.660	0.745	60	91	52	1	1006
Caiman	Rest/Wetland	0.660	0.188	4	6	-45	0	69
Caiman	Rest/Water	0.660	0.018	0	0	-14	0	5
Dale	Rest/Forest	0.849	0.936	81	95	13	1	2414
Dale	Rest/Water	0.849	0.012	1	1	-14	0	15
Dale	Rest/Wetland	0.849	0.050	4	4	-7	1	111
Anderson	Rest/Forest	0.707	0.881	65	92	18	1	2972
Anderson	Rest/Water	0.707	0.023	1	2	-6	1	56
Anderson	Rest/Wetland	0.707	0.094	5	6	-16	1	210

According to the analysis of the transit state, jaguars were more likely to move in wetland and water areas (Table 7).

**Table 7:** Measures resulting from mining by association rules between transit state and land cover.

Animal	Behavior State/Land Cover	SupA	SupB	Sup (%)	Conf (%)	Phi (%)	Lift	Occurrences (A∩B)
Alice	Transit/Forest	0.074	0.015	0	2	1	1	3
Picolé	Transit/Wetland	0.055	0.061	0	7	1	1	9
Picolé	Transit/Forest	0.055	0.070	0	4	-2	1	6
Fera	Transit/Wetland	0.028	0.098	1	31	12	3	39
Fera	Transit/Water	0.028	0.011	0	7	10	6	9
Fera	Transit/Forest	0.028	0.890	2	62	-15	1	78
Caiman	Transit/Water	0.078	0.018	1	11	21	6	15
Caiman	Transit/Wetland	0.078	0.188	2	22	2	1	29
Caiman	Transit/Forest	0.078	0.745	4	50	-16	1	66
Dale	Transit/Water	0.033	0.012	0	13	20	11	13
Dale	Transit/Wetland	0.033	0.050	1	24	16	5	24
Dale	Transit/Forest	0.033	0.936	2	61	-24	1	60
Anderson	Transit/Wetland	0.058	0.094	2	30	18	3	80
Anderson	Transit/Water	0.058	0.023	1	9	11	4	24
Anderson	Transit/Forest	0.058	0.881	3	60	-21	1	159

## Discussion

Our framework uses trajectory analysis and association rule mining [27] to provide statistical measures of correlation and dependence between associations and can be used to determine the relationship level between animals, their social interactions, and their interactions with other environmental factors based on their individual behavior and movement data. The higher the frequency of cooccurrence of these animals is, the greater the likelihood of interaction between them. This allowed us to describe transitions in the movement behaviors and social interactions of jaguars, contributing to a more detailed investigation of the species’ decisions to move at a fine scale.

The interactions between jaguars were explored through association rule mining that provided measures, such as the cooccurrence frequency (Sup), the probability of one animal occurring as a function of another animal’s occurrence (Conf), and the dependence (Lift) and correlation (Phi) between these animals. It is worth mentioning that association rule mining, which is commonly used to identify relationships between market basket products, effectively uncovered relationships between animals, as shown in this study. To validate the data mining results, the Cramer coefficient was also used to analyze the correlations between the animals; this algorithm is specifically designed for the study of correlations between categorical variables. The results of the association rules were similar to the results from the Cramer coefficient. Most adult jaguars of both sexes maintain individual territories, and individual interactions have been described for mating, male competition for females and carcass sharing in high-density areas [39–41]. We observed a large number of interactions in particular between the male Dale and the female Fera. We speculate that this is a mating pair, with the male Caiman trying to approach on some occasions. In fact, anecdotal reports indicate that females in estrus can attract several males, which can increase social interaction. Our data did not indicate the occurrence of a coalition, which is a common social behavior for lions that has been described once for jaguars [39].

In addition to the results obtained on social interactions between jaguars, the framework also provides information on the individual behavior of animals. With this information, it is possible to identify the time spent by jaguars in their daily activities, the times and periods of the day when the states of behavior occurred and their variations. We observed, in general, the foraging state was predominant between midnight and 8:00 AM. The resting was the predominant behavior state in the first months of the year. In our study site, these months represent the hottest period of the year and are also the flooding season, which are conditions that may limit the animals’ movement. The behavior state may also differ across the species distribution range. In human-modified landscapes, jaguars move long distances [4], and we may expect a higher frequency of transit states in these areas. Human activities induce shifts in the natural pattern of animal activity, increasing nocturnality [42]. In this way, in areas with more human activity, jaguars can increase their foraging and transit behavior states at the night while resting during the day. Tracking jaguars in this kind of environment is crucial for addressing differences in their activity patterns.

Our approach also allowed us to identify the space occupied by individual animals along with the space shared with other animals over months and years and according to variations in behavioral states. We also associated animal behavioral states with the landscape. Forests represent an important habitat for jaguars, which used this landscape mostly for resting but also for transit and foraging. A previous study reported the importance of forest coverage in jaguar habitat selection; animals in highly forested areas strongly avoided nonforested areas [43]. Associating the behavior state with the landscape structure is key to understanding how the jaguar adjusts its movement behavior in human-modified landscapes.

Unlike the other approaches found in the literature, this framework uses the association rules mining to analyze the social interactions between typically solitary animals as jaguars and between behavioral states and the environmental factors that surround it. In addition, it provides information about the individual behavior of these animals.

In short, this framework allows the analysis of animal movement and presents a set of statistical measures about individual animals and their interaction with conspecifics and the environment, which enables researchers to:

- classify animal behavior according to states such as rest, foraging and transit;
- determine the distance between animals;
- identify the social interaction probability between animal pairs;
- identify the interaction between animals and environmental factors;
- identify the periods (day/night) that most frequently correspond with the animal’s behavioral states;
- identify the time when the animal most frequently performs foraging, rest, or transit activities;
- identify the duration of time the animal spends performing foraging, resting or transit activities;
- identify the home range by animal or by behavioral state, as well as the overlapping home ranges between animals over time (month/year), and monitor the evolution of the use of space by the animal over time and even by behavioral state over space and time; and
- identify gaps in data on animal movement at certain times of the year.

Therefore, the knowledge obtained through spatiotemporal analysis of animal movement is essential. The valuable information this analysis provides allows us to understand changes in animals’ lives, animal behavior, how animals use space over time and the relationships of individual animals to other animals. This understanding can be used to guide researchers and public agencies in decision-making.

## Supporting information

S1 Fig Average durations of behaviors of the jaguar Picolé. The graph shows each jaguar behavior state as a line. The results indicated that Picolé foraged, on average, for two hours a day between January and April and for one hour a day in March 2015.
(TIF)

S2 Fig Jaguar behavior by hour intervals. The graph shows the time intervals during which Picolé foraged most frequently in 2015. The circle represents the start time and the line represents the duration, thus indicating the start and end of the period. From January to May 2015, the results indicated that Picolé foraged more from 0 a.m. to 8 a.m. than during other time periods, with some variations over the months.
(TIF)

S3 Fig Frequency of behavioral states of the jaguar Picolé by period (day/night). The frequency of each jaguar behavior over the periods of the day (day, night, or day/night) was calculated based on the times of occurrence of the behavior. The day/night period represents the occurrences that started during the day and ended at night. The graph shows the frequency of each of the jaguar Picolé’s states of behavior over the months from January to May 2015, indicating a higher number of foraging occurrences at night. In February and March, there was an increase in occurrences of night foraging, followed by a decline in the months of April and May 2015.
(TIF)

S4 Fig Picolé home range map: (A) by month and year and (B) by state of behavior, month, and year The map provides information about the variations in the space occupied by the jaguar Picolé over months and years (Sup4A) and by behavior state and month of the year (Sup4B). For example, Picolé occupied a certain area in January 2015, migrated to a new area throughout February and remained in this area during the following months.
(TIF)
