# Supplementary figures and images for: Jaguar movement behavior: using trajectories and association rule mining algorithms to unveil behavioral states and social interactions
Source: PLoS One. 2021 Feb 4;16(2):e0246233. doi: 10.1371/journal.pone.0246233 (PMC7861389; doi:10.1371/journal.pone.0246233)

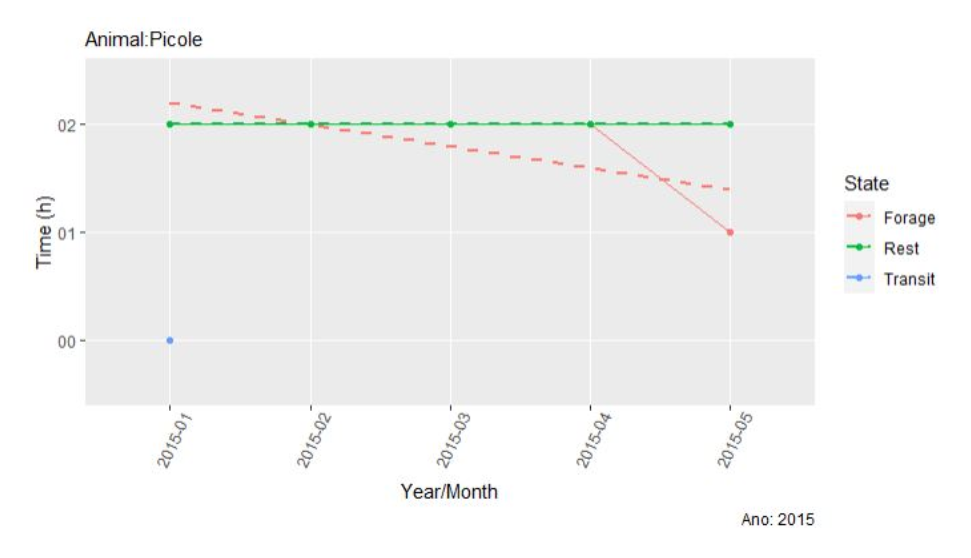

Supplement: S1 Fig — The graph shows each jaguar behavior state as a line. The results indicated that Picolé foraged, on average, for two hours a day between January and April and for one hour a day in March 2015. (TIF) [file pone.0246233.s001.tif]

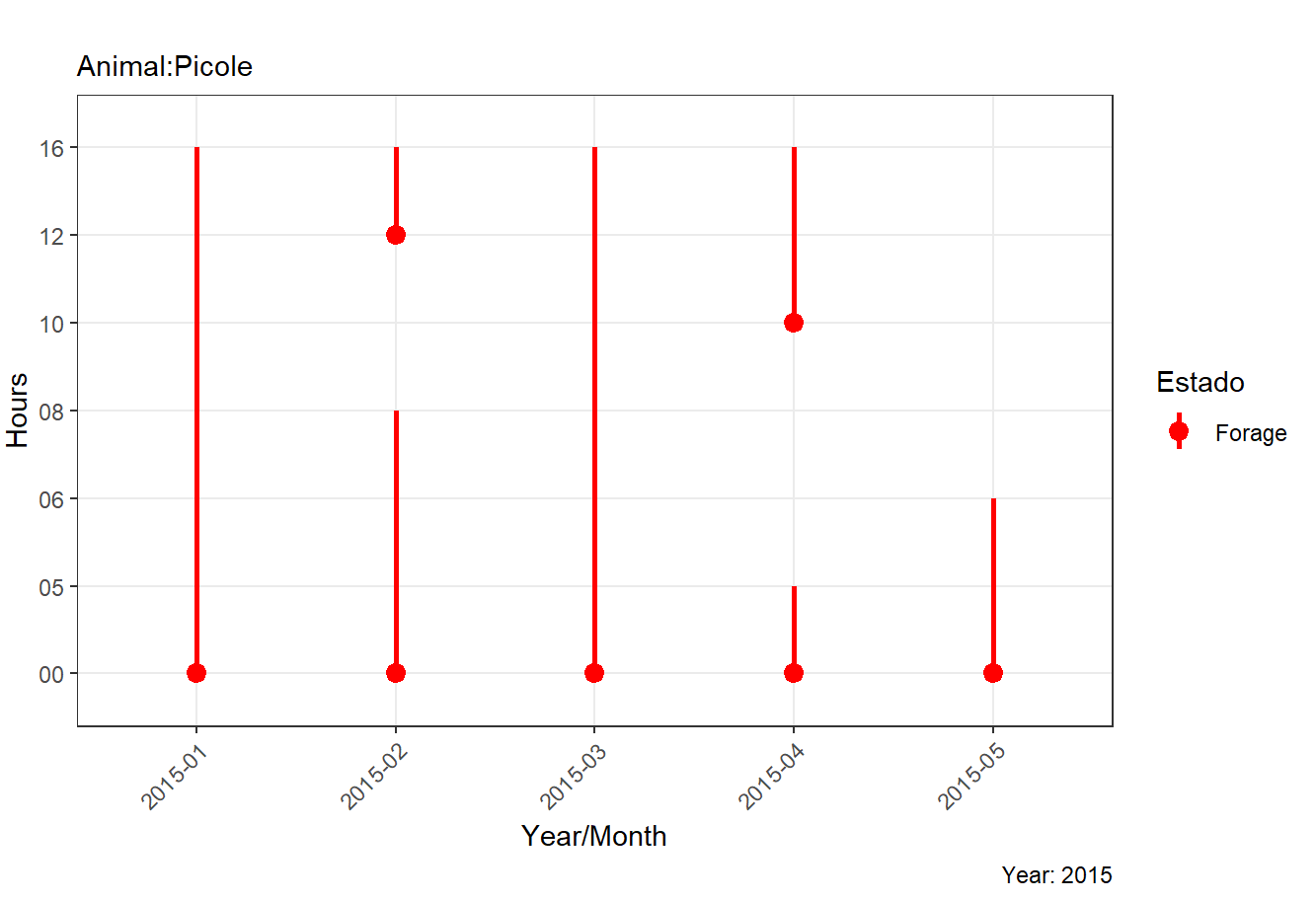

Supplement: S2 Fig — The graph shows the time intervals during which Picolé foraged most frequently in 2015. The circle represents the start time and the line represents the duration, thus indicating the start and end of the period. From January to May 2015, the results indicated that Picolé foraged more from 0 a.m. to 8 a.m. than during other time periods, with some variations over the months. (TIF) [file pone.0246233.s002.tif]

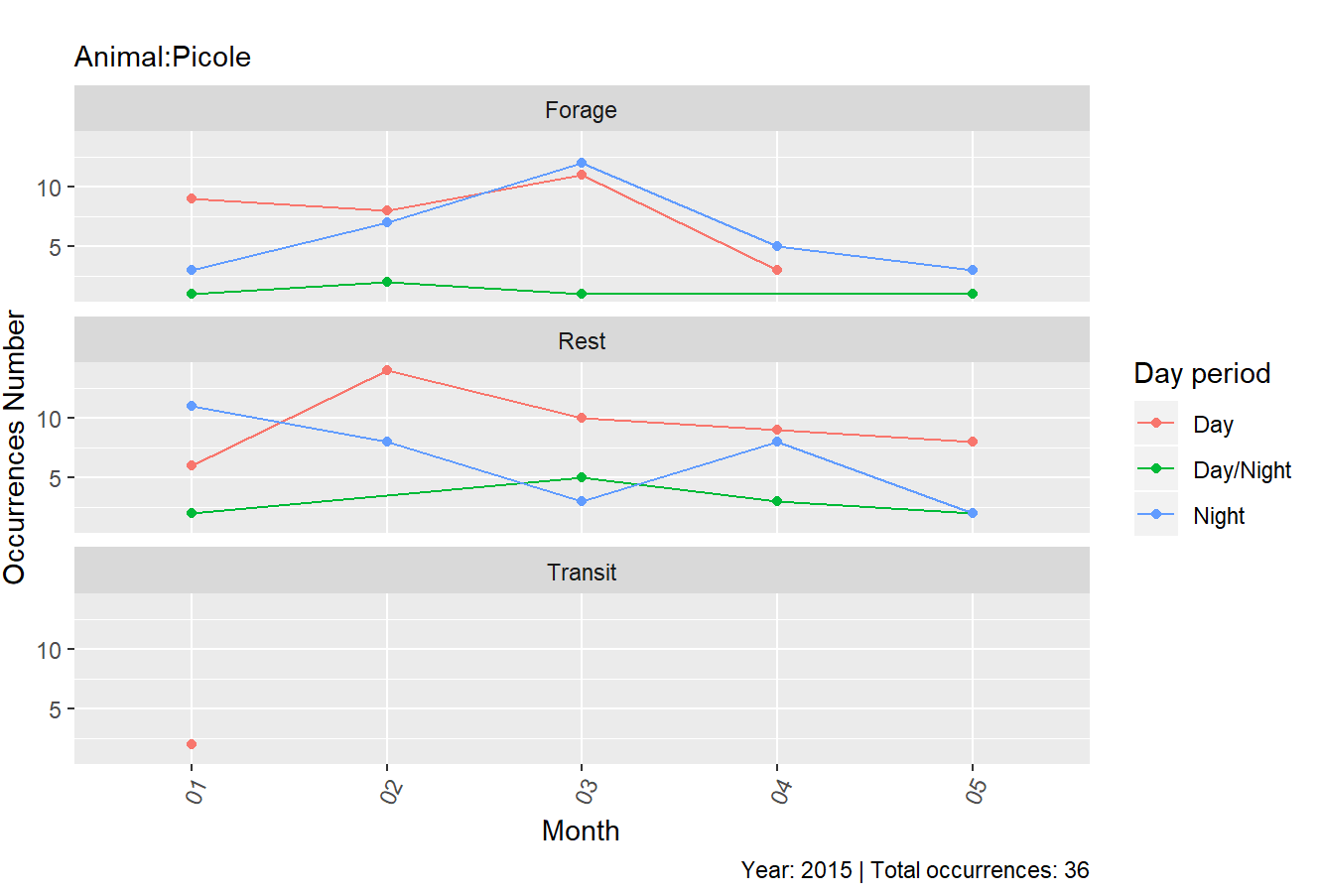

Supplement: S3 Fig — The frequency of each jaguar behavior over the periods of the day (day, night, or day/night) was calculated based on the times of occurrence of the behavior. The day/night period represents the occurrences that started during the day and ended at night. The graph shows the frequency of each of the jaguar Picolé’s states of behavior over the months from January to May 2015, indicating a higher number of foraging occurrences at night. In February and March, there was an increase in occurrences of night foraging, followed by a decline in the months of April and May 2015. (TIF) [file pone.0246233.s003.tif]

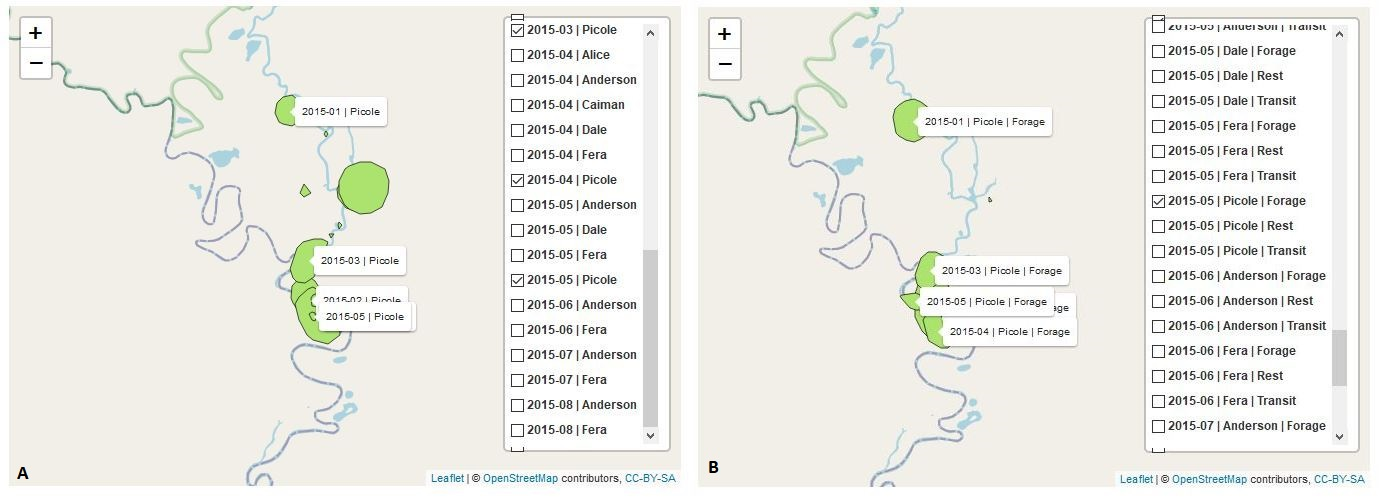

Supplement: S4 Fig — Picolé home range map: (A) by month and year and (B) by state of behavior, month, and year The map provides information about the variations in the space occupied by the jaguar Picolé over months and years (Sup4A) and by behavior state and month of the year (Sup4B). For example, Picolé occupied a certain area in January 2015, migrated to a new area throughout February and remained in this area during the following months. (TIF) [file pone.0246233.s004.tif]
